# Influences on prescribing in borderline personality disorder: insights from health care professionals: a qualitative interview study

**DOI:** 10.1186/s12888-026-08104-y

**Published:** 2026-04-27

**Authors:** Joshua Confue, Ian Maidment, Matthew Jones

**Affiliations:** 1 University of Bath & Lincolnshire Partnership Foundation Trust, Lincoln, UK; 2 Aston University, Birmingham, UK; 3 University of Bath, Bath, UK; 4 Lincolnshire Partnership NHS Foundation Trust, Lincoln, UK

**Keywords:** Borderline personality disorder, Prescribing, Qualitative study

## Abstract

**Background:**

Borderline Personality Disorder (BPD) is a complex psychiatric condition that significantly impacts not only individuals but also wider society. Despite the absence of licensed medication for BPD, there is substantial evidence of high rates of prescribing and polypharmacy within this population. This has raised concerns, given the potential adverse effects of psychotropics. This study aimed to explore the potential factors influencing prescribing decisions in patients with BPD.

**Method:**

We conducted semi-structured interviews with healthcare professionals involved in prescribing for individuals with BPD. Participants included doctors, non-medical prescribers, and pharmacists from both primary and secondary care settings. Data was analysed using thematic analysis utilising both inductive and deductive approaches.

**Findings:**

Twenty interviews were completed, generating five key themes believed to influence prescribing decisions. These themes indicate that prescribing occurs at least in part to manage specific symptoms; address perceived risks; maintain or strengthen the therapeutic relationship; compensate for the lack of alternative treatment options; and respect patient autonomy and choice.

**Conclusions:**

Participants highlighted the complexities involved in treating individuals with BPD, indicating that prescribing decisions were often more than simple risk-benefit assessments of the prescribed medication. In particular, they highlighted the challenge of balancing risks while maintaining a therapeutic relationship. Additionally, the findings indicate that a patient's preference for medication may lead prescribers to embrace a greater risk tolerance when prescribing.

**Trial registration:**

The study was registered with and received a favourable outcome from the Human Research Authority (IRAS 330510) and was approved at the London - Camberwell St Giles Research Ethics Committee (REC) on the 12th of December 2023.

**Supplementary Information:**

The online version contains supplementary material available at 10.1186/s12888-026-08104-y.

## Introduction

Borderline Personality Disorder (BPD), sometimes referred to as Emotionally Unstable Personality Disorder (EUPD), denotes a mental health condition characterised by pervasive patterns of instability regarding interpersonal relationships and self-image, alongside marked impulsivity [1]. BPD has an estimated prevalence range of between 0.7% and 5.9% [2] in the general population. Notably, BPD presentation is substantially higher among patients under psychiatric services, with patients with BPD constituting 15–28% of service users [3]. Despite significant morbidity and presentation, there are no medications licensed for BPD. This is reflected in most major guidelines, including those of the American Association of Psychiatrists [4] and the National Institute for Health and Care Excellence (NICE) of England [5], concurring that there is insufficient evidence to recommend the use of medication in the treatment of BPD [6].

However, previous studies have shown high levels of psychotropic prescribing within this cohort. A Massachusetts 16-year follow-up study [7] estimated that 40% of patients with BPD are prescribed three or more psychotropic medications during their care. This estimate is supported by a 2015 inpatient study conducted across Europe, which found that over 10 years, 70% of BPD patients were prescribed antipsychotics, antidepressants, or a combination thereof [6]. Congruently, a 2025 Swedish study has indicated a potential increase in medication levels among individuals diagnosed solely with PD between 2011 and 2020 [8]. This evidence paints a picture of significant levels of psychotropic prescribing in BPD despite recommendations.

While there is limited evidence of the benefits of medication in BPD, the potential harms of psychotropic medications are well evidenced. Multiple psychotropics have been associated with metabolic disorders, including, but not limited to, diabetes, dyslipidaemia and metabolic syndrome [9]. This risk is most pronounced with the use of second-generation (atypical) antipsychotics, with a 2021 systematic review of population-based studies finding that no drug in this class is devoid of risk [10]. In addition to metabolic risks, all antipsychotics also carry the risk of movement disorders such as dystonia and akathisia. These established risks and poorly defined benefits raise the question of what is driving the levels of prescribing observed in BPD patients.

A consistent element of the prescribing process is the assessment of possible benefits of a medicine against its potential harms; yet, with BPD, the poor evidence base around medication, coupled with disagreements around diagnosis and categorisation [11], makes this a more complicated endeavour.

In addition, there are also other, more nuanced influences in prescribing. A 2018 review identified 33 frequently highlighted factors, including a medic’s personal attributes, medication costs, and patient preferences [12]. While evidence is limited, this may indicate that prescribing influences can differ across clinical settings, highlighting the importance of investigating BPD specifically to understand how these influences impact treatment approaches.

A 2025 systematic review of the factors that influence prescribing in BPD identified several demographic characteristics linked to higher levels of prescribing, notably patients’ older age and the presence of comorbid conditions. Of particular note, the authors also highlighted two key themes that impact the prescribing process: the healthcare professional (HCP)-patient relationship and the difficulties navigating care pathways [13]. However, the included studies were often limited to single settings, and did not include non-medical prescribers, a growing group of HCPs in England and other regions [13].

The current study, therefore, aimed to assess the perspectives of HCPs, both medical and non-medical, involved in the prescribing process for BPD across multiple primary and secondary care services in the United Kingdom (UK), and explore the factors influencing their prescribing decisions.

## Methodology

### Study design

Qualitative semi-structured interviews were used to explore the perspectives of HCPs in depth. The study was registered with and received a favourable outcome from the Human Research Authority (IRAS 330510) and was approved at the London - Camberwell St Giles Research Ethics Committee (REC) on the 12th of December 2023. The study was conducted in 2024, and informed consent was obtained from all participants. All procedures performed in the study were in accordance with the ethical standards of the University of Bath and the 1964 Declaration of Helsinki and its later amendments.

Given the limited literature regarding influences on prescribing in BPD, the research team selected Agency Theory to guide the study. Agency Theory posits that an “agency relationship” arises when principals delegate authority to agents who act on their behalf [14]. In the paradigm, the prescriber acts as the agent, with the patient acting as the principal [14]. This is because the principal (patient) delegates the selection of medications for their treatment to the agent (prescriber), constituting a principal-agent relationship. This model also aligns with the idea of the HCP-patient relationship as highly important to the process, a consistent finding of existing literature [15].

**Fig. 1:**
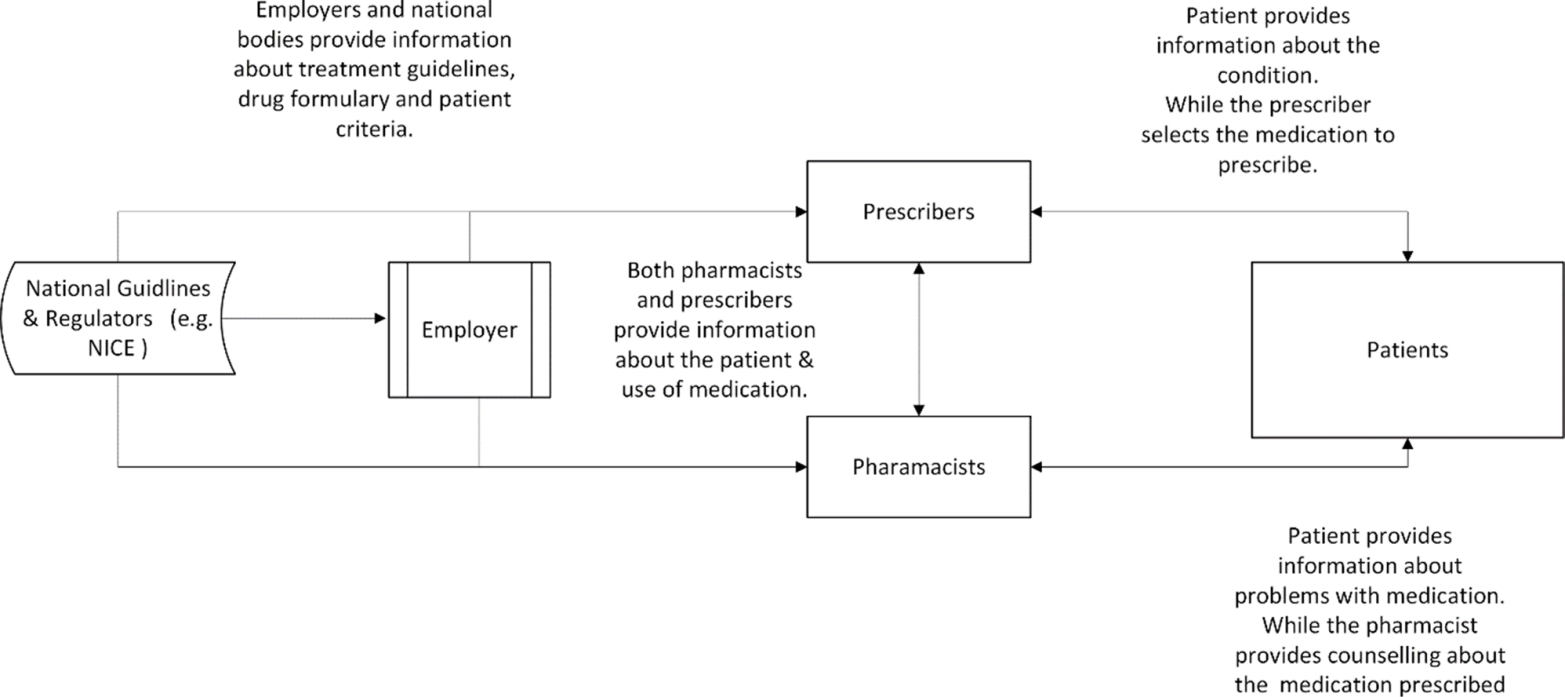
Mapping of agency- princpical relatioships in prescriing for BPD adapted from [16], original diagram

During the initial stages of the study, Agency Theory was applied to map the relationships involved in prescribing decisions, including relevant control mechanisms (see Fig. 1). In particular, three of Agency Theory’s fundamental assumptions are that the principal and the agent possess incomplete information, have differing goals and risk profiles. Therefore, the interviews aimed to explore and clarify these goals, as well as identify the information available to and utilised by both parties.

### Recruitment and sampling

Participants were eligible for inclusion if they were doctors, non-medical prescribers, or pharmacists who had cared for someone with BPD and been involved in the prescribing of medication for BPD in Great Britain within the last year. Medical and non-medical prescribers were selected due to their direct authority to diagnose and prescribe within their area of clinical competence. While, pharmacists were included due to their role, in the UK, in providing advice on the safe and effective use of medicines. 

A target sample size was based on existing published qualitative studies of HCPs and to account for a range of types of HCP [17]. The aim was therefore to conduct between 15 and 20 interviews. In line with the principles of information power [18], interviews continued until the data collected was deemed sufficient to support valid and meaningful thematic analysis. The study was promoted through five NHS Mental Health Trusts in England, professional bodies, special interest groups, and ‘snowballing’ via previous participants. Participants subsequently approached the research team to express their interest in participating.

### Data collection and analysis

All interviews were conducted by JC in private places or online, digitally recorded, and subsequently transcribed by the interviewer (see COREQ checklist for additional information, Appendix-A) [19]. Following written, informed consent, interviews typically lasted 30–45 min.

A semi-structured interview schedule (Appendix-B) was developed specifically for this study using the findings of a recent systematic review [13], and refined through engagement with both HCPs and patients with BPD. This involved one-to-one sessions to discuss the project and allow individuals to comment and critique the schedule. This engagement highlighted some of the complexities inherent to shared decision-making and the HCP-patient relationship. The guide included questions relating to choices of medication, duration, efficacy, assessment of response to treatment, education and training, as well as self-identified influences on prescribing decisions.

A deductive and inductive thematic analysis was used to generate codes and themes, as outlined by Braun & Clarke [20–22], based primarily on the data’s semantic content. Interviews were transcribed and read multiple times to achieve familiarity with the content. Throughout the project, the primary researcher maintained a reflexive journal to document assumptions and interpretations, revisiting these throughout the analysis to provide an awareness of interpretive decisions.

Agency Theory informed both our analytic process and the interpretive lens applied throughout the thematic analysis of interview transcripts. Overall, Agency Theory did not inform the generation of primary codes. Instead, it provided the scaffolding for the deductive component of our analysis, guiding the research team’s exploration of participants’ accounts. It was primarily applied at the interpretive stage, shaping the construction, organisation, and presentation of themes derived from the coded data. Specifically, the team examined established sources of tension within principal–agent relationships, including goal divergence, differences in risk tolerance, and information asymmetries.

Manual coding of the first three transcripts (by JC) was followed by a team review to establish consensus on initial codes, before progressing. This process involved generating as many relevant codes as possible and refining them through discussion. The team then searched for potential themes and subthemes, organising codes into broader categories utilising triangulation through diagramming to facilitate identification of theme connections. Themes were subsequently reviewed against the coded data and the entire dataset to ensure completeness and consistency.

Once refined, themes were clearly defined and named. A mind-mapping exercise was used to facilitate discussion and ensure that the labels captured the essence of each theme and were representative of the dataset. NVivo software supported the organisation and management of transcripts, but did not conduct the analysis itself [19].

## Results

Twenty interviews were completed with a range of pharmacists, medical and non-medical prescribers (Table 1). Analysis generated five main themes describing influences on prescribers’ decisions for prescribing in BPD (Fig. 1): Symptomatic Treatment; Risk; Validation and the Therapeutic Relationship; System Constraints; and Patient Choice.

**Table 1:** Demographics of participants

	Role	Setting	Age	Ethnicity	Gender
HP1	NMP (Pharmacist)	Primary Care (In a prescribing role)	50–59	White (British)	Female
HP2	NMP (Nurse)	Primary Care (In a prescribing role)	50–59	White (British)	Male
HP3	NMP (Pharmacist)	Primary Care (In a prescribing role)	50–59	White (British)	Female
HP4	Consultant	Secondary Care	30–39	White (Italian)	Female
HP5	Consultant	Secondary Care	30–39	White (British)	Male
HP6	NMP (Nurse)	Primary Care (In a prescribing role)	30–39	White (British)	Female
HP7	Consultant - Nurse	Secondary Care	40–49	White (British)	Female
HP8	ACP	Secondary Care (In a prescribing role)	40–49	White (British)	Female
HP9	Consultant	Secondary Care	50–59	White (British)	Female
HP10	Consultant	Secondary Care	60–69	Asian or Asian British (Indian)	Female
HP11	Specialist Grade Doctor	Secondary Care	30–39	Asian or Asian British (Indian)	Male
HP12	NMP (Nurse)	Primary Care (In a prescribing role)	30–39	White (British)	Female
HP13	NMP (Pharmacist)	Primary Care (In a prescribing role)	40–49	Asian or Asian British (Bangladeshi)	Female
HP14	Pharmacist (Non-Prescribing)	Primary Care (In a prescribing role)	20–29	White (British)	Male
HP15	NMP	Primary Care	50–59	White (British)	Male
HP16	NMP	Secondary Care	ND	ND	ND
HP17	Specialist Grade Doctor	Secondary Care	20–29	Asian or Asian British (NOS)	Female
HP18	Pharmacist (Non-Prescribing)	Secondary Care (Ward Pharmacist)	20–29	White (British)	Male
HP19	Consultant	Secondary Care	50–59	Asian or Asian British (Indian)	Male
HP20	NMP (Pharmacist)	Primary Care	30–39	White (British)	Female

**Foot Note**: NssMP- Non-Medical Prescriber, ACP- Advanced Clinical Practitioner, ND- Not Disclosed, NOS- Not Otherwise Specified

### Prescribing for symptomatic treatment

Participants reported different approaches and strategies around medication in BPD. All participants spoke about the potential benefit of medication for some patients in providing symptomatic relief:

*…[symptoms] that people struggle with the most*,* so I try to treat those top symptoms Participant*-*HP7*

*Yeah*,* 100%*,* at least with the consultant I work with at the moment*,* is definitely prescribing for symptoms*,* it’s not for the diagnosis Participant*-*HP17*

Where participants felt medication could be utilised for symptoms, it was predominantly focused on four main aims:

- providing relief or support to engage in or utilise other strategies, such as psychologically-based interventions:

*[medication] sometimes allows them to have some energy to then engage in therapy and do things differently Participant-HP4*

b) providing immediate relief from particular emotional distress and/or enabling sleep:

*…some relief from the stress you’re experiencing at the moment Participant-HP8*

*…a lot of the drugs are sedating*,* aren’t they*,* so if you’re quite distressed*,* sedation will help Participant-HP9*

c) providing an ongoing ‘buffer zone’ (psychological or emotional space to enable individuals to manage difficult emotions) to support recovery in less acute cases, for example, after initial treatment success or following the resolution of a crisis:

*…the big question I always ask is: can medication act as a buffer*,* you know*,* hence I would just leave a low dose neuroleptic Participant-HP19*

d) management of co-morbid conditions:

*I am more likely to prescribe*,* so [for] depression*,* persistent anxiety*,* generalised anxiety*,* we know the drugs work in those situations*,* and those comorbidities*,* and those symptoms Participant-HP19*

Notably, some participants felt this benefit was only for co-morbid symptoms or conditions, particularly those symptoms associated with recurrent depression, anxiety and panic disorders (a particularly complex area, reflecting the difficulties faced by individuals managing multiple psychiatric or medical conditions simultaneously):

*Medication cannot change the symptoms*,* what we would call the BPD symptoms… I think they can help on some co-morbid symptoms*,* like you know*,* anxiety*,* hallucinations*,* depressive episodes Participant-HP4*

The reasons professionals believed in the efficacy of these medications for symptom relief encompassed three main areas: previous experience, colleagues, and evidence in other patient groups:

*The majority of learnings from my training obviously*,* experience*,* but all of us are… professionals and supposed to be daily learners*,* so I definitely learned from my colleagues*,* the team… Participant-HP11*

This represents a weighting by HCPs of their own experiences over research evidence from systematic reviews. When explored, most participants assessed the efficacy of medication largely through the patients’ subjective descriptions and narratives. Despite this, multiple participants acknowledged a possible large placebo effect:

*[How about assessing efficacy? ] “…it depends*,* it’s such a subjective [thing]*,* it depends on what they’re telling me Participant-HP13*

*I think the way the decisions are made about prescribing*,* or that perception of response to treatment … is all really*,* really subjective*,* and there’s a term that one of my colleagues used which is ‘vibe based’ Participant-HP18*

### Prescribing around risk

All participants spoke about risk, and the idea of risk and benefit as part of the prescribing process was prominent in multiple conversations. Indeed, when asked, risk was defined by multiple participants as the single largest factor in their prescribing decisions:

*Harm is probably a big thing. So if I think giving medication could perpetuate*,* or increase*,* or introduce harm to the patient*,* … it may be from*,* you know*,* physical health condition making that worse*,* side effects making that worse*,* overdose risk*,* self-harming risk*,* making that worse. That’s a big element. Participant-HP12*

The definition of risk, however, was different for participants, with considerable variation in the focus, direction, and prioritisation of risks. For some HCPs, the risk of suicide, self-harm, and harm to others was particularly concerning, especially where it was felt to result from an unmanaged psychiatric condition. The need to reduce the risk of such an event was a prominent reason to prescribe. Conversely, the risk of overdose, side effects, and interactions, both with other medications and disease states, were sometimes seen as reasons not to prescribe:

*Kind of*,* self-harming is probably number two. The thing we want to reduce [is] that tension and urge and impulsivity regarding harming themselves*,* cutting and burning*,* and swallowing objects*,* and all those. They needed some level of treatment*,* obviously. Participant-HP10*

Participants also highlighted the idea of the risk of prescribing leading to psychological dependence or physiological addiction, which was particularly notable in the case of benzodiazepines but also with other classes of psychotropics. Fundamentally, a concern was expressed around the risk of the formation of a ‘psychological crutch’ that may deprive people of agency, which, in turn, may be a detriment to the patients’ long-term recovery:

*They’ll see the medication as a crutch*,* instantly resolving the anxiety. That sounds awful*,* I feel I have a duty of care to make sure they are not addicted to their medication Participant-HP3*

*One of the negatives of [prescribing] is that it takes away that*,* that sense that you have the solution inside you Participant-HP5*

### Prescribing for validation and the therapeutic relationship

Participants spoke about the role of validation and trust, and their importance, in enhancing therapeutic outcomes. Participants reported the importance of patients being “heard” and “understood” by HCPs as vital in maintaining the therapeutic relationship, and how prescribing could facilitate this process.

A positive therapeutic relationship being one of the most important aspects of treatment was a prominent reflection:

*Otherwise*,* if the therapeutic relationship breaks down*,* nothing will work. Nothing will work. Participant-HP19*

Participants reported that decisions not to prescribe could lead to negative experiences and the perceived invalidation of patients’ concerns, in turn damaging this critical therapeutic relationship:

*If you don’t think medication will be useful in the case*,* you don’t want the person to feel like they’re not being listened to or dismissed Participant-HP15*

It is worth noting that this therapeutic relationship can extend beyond the patient and HCP to the family or support network of the patient, particularly in younger individuals or those lacking capacity. Capacity being defined as the ability to use and understand information to make a decision and communicate any decision made [23]. In the UK, the Mental Capacity Act 2005 provides the framework for people who may lack mental capacity:

*… one of my 18-year-old patient[s]*,* the mum (of a detained patient) said every week*,* ‘why is she not on an antidepressant’ Participant-HP5*

### Prescribing owing to system constraints

All participants described issues with limited access to treatment options other than medication (particularly long-term psychological therapies) and how this had impacted patient outcomes:

*You don’t have an IAPT [Improving Access to Psychological Therapies] service that deals with personality disorders … I think the resources in this county*,* and probably nationwide*,* in terms of emotional dysregulation and those difficulties*,* are a challenge… Participant-HP2*

Participants reported systemic barriers in accessing support for patients and a need for an accessible range of diverse interventions. In particular, participants often described frustration with a ‘one-size-fits-all’ approach to mental health support. For example, a requirement for a formal diagnosis of personality disorder to access a particular intervention:

*We did run a DBT [Dialectical Behaviour Therapy] service*,* and it was successful … it’s hard to get a person into a community mental health team [to] have a psychological assessment and start therapy. There’s only a small number of patients who will get that. Participant-HP7*

*There are not a lot of psychological services they can access for support. The local talking therapies services all exclude patients with BPD. Participant-HP1*

*…they have to have a diagnosis of personality disorder to be able to access that [psychological therapy DBT] Participant-HP20*

This reflects both a perceived lack of services but also significant differences in access criteria for mental services across the country. Overall, this left some participants feeling trapped in a medication-only paradigm due to a lack of accessible alternatives:

*No*,* to be honest with you. No*,* I think it’s cheaper and easier to prescribe that citalopram than have the proper therapy-sation with the right support… Participant-HP16*

### Prescribing for patient choice

Participants working across all settings spoke about the role of autonomy and patient choice in psychiatric treatment and prescribing; the right to choose, or decline, medication was prominent. Emphasis was placed on the right to take risks, where the individual understood the benefits and the risks and had capacity, even when, in the HCP’s opinion, the decision being made was sub-optimal:

*…people do a lot of research online*,* social media platforms*,* where they’ll be involved in that research via forums*,* TikTok… and they’ll come in… and ‘I wanna try that and that’ Participant-HP6*

*There is always a little bit of give and take*,* but as long as it’s reasonable because nothing is set in stone here and it’s about what works and if what the client is asking for is reasonable Participant-HP19*

Overlapping somewhat with the third theme of the influence of the therapeutic relationship, participants reflected that prescribing is a ‘joint journey’, here focusing more closely on the concept of patient agency and the right to personal choice:

*Success rate is likely to be higher if the patient is involved and engaged in the decision. They’re more like[ly] to keep going longer*,* tolerate side effects*,* as … they’ve chosen to do it. But also a little bit for me ‘cause as they’re choosing*,* we are embarking on this journey together. [It] is not just me saying this might be a good idea. So*,* if something goes wrong*,* we did this together. I don’t know… it sounds bad when you say [it] out loud. It’s not to alleviate my stress but because it feels like that’s a better way of working with this patient group. Participant-HP20*

This also suggests a belief in a shared responsibility when a patient chooses to pursue an approach.

## Discussion

### Statement of principal findings

**Fig. 2:**
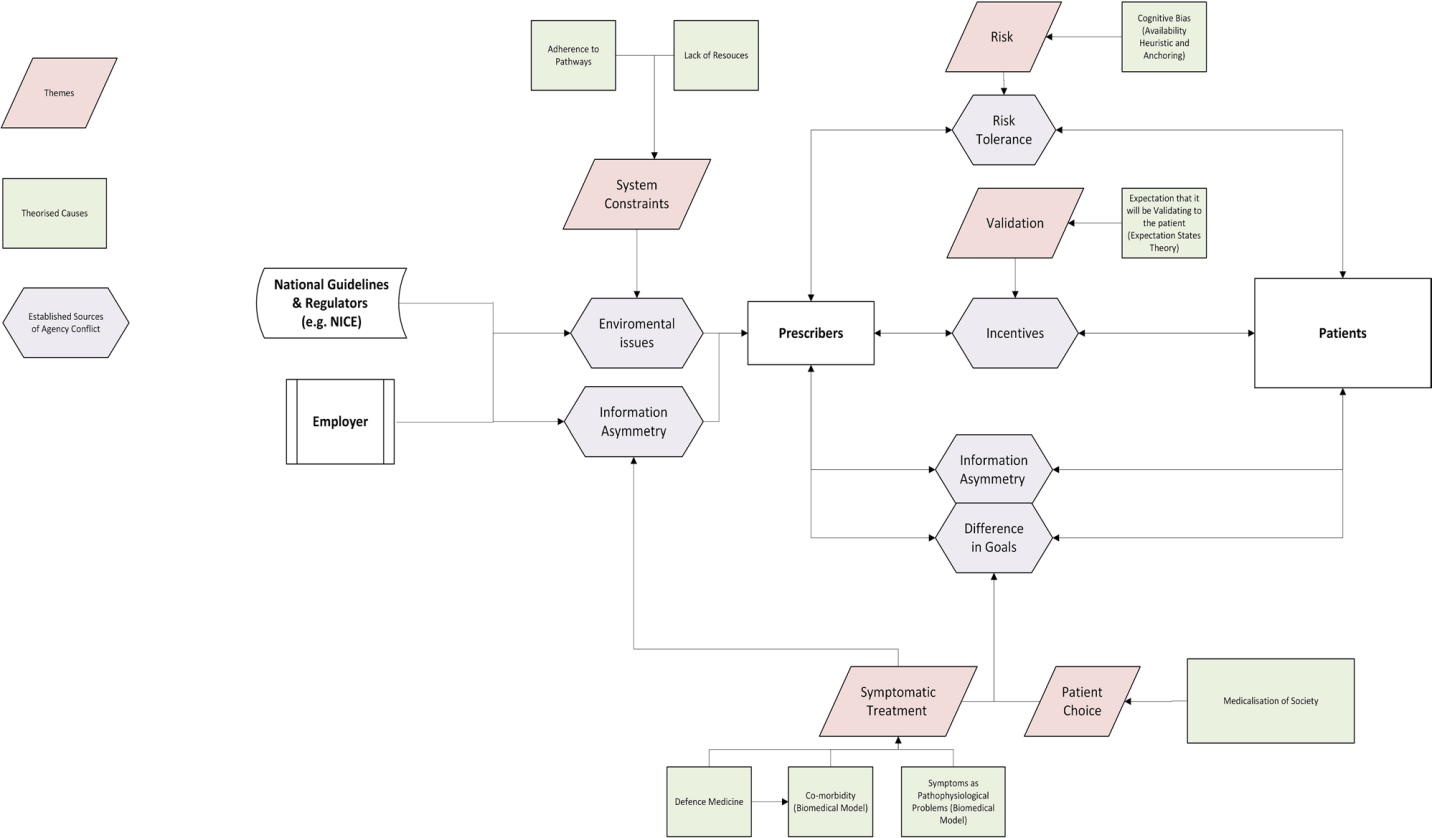
Map of generated themes and sources of agency conflict in prescribing relationships

**Fig. 3:**
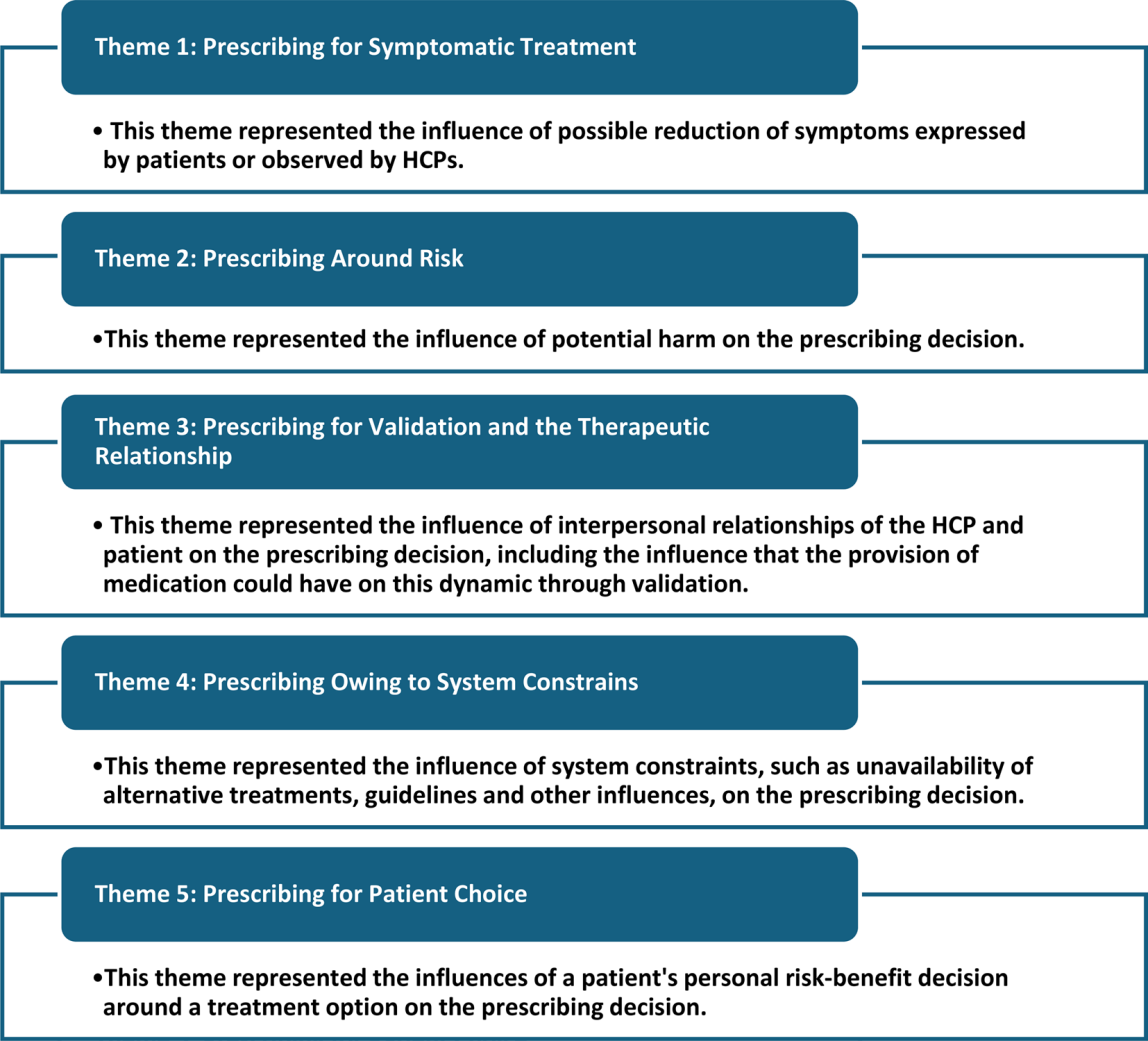
Description of five main themes generated

This study indicates that medication is prescribed to BPD patients for overlapping reasons: to treat symptoms; address perceived risks; build or maintain a therapeutic relationship; work around a lack of alternative options; and respect patients’ right to choice. These complex themes (see Figs. 2 and 3) describe the factors that influence prescribing decisions beyond simple risk-benefit assessments of the medication.

### Findings in relation to other studies

The primary limitation of the current body of research to date, lies in its limited scope, focusing on a single type of HCP, alongside its overall limited size. This study included 20 participants compared to only 60 participants in total for the four previous studies on this topic [13]; it was also the first to include Non-Medical Prescribers (NMPs).

Some of the reported core influences identified are reflected in the existing literature. The importance of the therapeutic relationship and how medication can affect this dynamic has been highlighted by existing research [24–27]. In addition, previous studies have highlighted symptomatic treatment [26–28] and barriers in navigating healthcare systems [25, 26] as additional influences on the prescribing decisions of HCPs. Notably, some previous research has downplayed the influence of patient choice and risk, for a greater focus on relational prescribing [24] .

Existing evidence suggests that prescribing practices for BPD may be shaped by demographic variables, with gender and age, for example, correlated with the likelihood and type of medication prescribed [13]. While these concepts were not self-reported by participants in this study as influences on their decisions, this does not mean that they do not have an impact. They may subconsciously shape prescribers’ decision-making.

### Symptomatic treatment

Theme 1 aligns with patterns of symptom-based prescribing identified in other conditions. A 2007 examination of psychotropic use in PTSD found substantial prescribing unrelated to the diagnosis, likely the result of symptom-specific treatment [29]. This idea is supported by a 2017 study, which examined outpatient psychiatric prescribing and found that symptoms predicted medication prescribing better than DSM diagnoses [30].

A belief in the ability of medication to provide symptomatic relief outside the management of co-morbidities represents an asymmetry of information between HCPs, governance structures and professional bodies. The cause of this asymmetry is unclear however, in this study, some participants reported a belief in the efficacy of psychotropics in treating symptoms shared by BPD and other conditions, defending the view that medication can be “transdiagnostic” (effective across conditions with shared symptomology) [30]. We theorise that differing epistemological orientations contributes to the information asymmetry. Some prescribers appear to give greater weight to personal experience, patient beliefs, and transdiagnostism; than those who write guidelines. This divergence may reflects the variation in purposes between those making individualised clinical decisions versus broader recommendations (guidelines), resulting in a shift in how evidence is interpreted and valued. This view is likely bolstered by regulatory approval being given for the use of specific medications in the treatment of multiple psychiatric conditions. 

The framework through which clinicians define BPD strongly shapes both prescribing practices and therapeutic engagement. One explanation for why participants prescribe for symptoms is that that they position symptoms as pathophysiological problems amenable to pharmacological intervention, this view may be rooted in a biomedical interpretation [31]. 

Yet, participants across disciplines consistently reported adopting a biopsychosocial perspective, emphasising the interplay of biological, psychological, and social factors, with particular attention to trauma. This focus on trauma may reflect the growing influence of trauma-informed pathways in the UK [32]. 

Another intertwined aspect of symptomatic relief was prescribing for comorbid conditions. It has been suggested that comorbidity is often used to justify the use of medication [11]. While not directly described in the interviews, it is not unreasonable to hypothesise that elements of Defensive Medicine may result in clinicians prescribing for co-morbid conditions to enable proactive intervention while complying with guidelines in uncertain situations [33]. Moreover, this desire to use medication to provide symptomatic relief reflects a broader cultural shift towards the medicalisation of distress and suffering [34, 35].

### Risk

Theme 2 identified the influence of risk. Divergence in risk tolerance is a common source of conflict in principal-agent relationships. While NICE reports that the probability of benefitting from the prescribing of psychotropic is too low, the evidence from this study and others suggests that at least some HCPs disagree with this. Despite growing awareness of the risks associated with psychotropic drugs, from benzodiazepines to antidepressants [36, 37], prescribing rates for this population remain high.

We suggest that this divergence risk tolerance arose in part because clinicians felt disproportionately accountable for certain adverse outcomes. Their perceived personal responsibility may have led to them placing less emphasis on the adverse effects of medications while attributing more emphasis to even the possibility of symptomatic improvement. Participants in this study clearly articulated the possibility of harm if medication is not used, such as the potential for escalation in symptoms, self-harm, hospital admission, invalidation of the patient, and breakdown of the therapeutic relationship. By comparison, harms arising from treatment, aside from the risk of overdose, were generally described as more remote. They were also often more nebulous, perhaps leading to them being less prominent factors in prescribing.

This appears to occur for two reasons: the focus on immediate safety and the siloed nature of healthcare. It has been previously suggested that the risk of side-effects, particularly metabolic syndromes, is a less imminent concern at the time of prescribing and therefore receives less weighting. A 2024 study found that psychiatrists reported unrealistic expectations around the prevention of adverse outcomes and that prescribing represented a way to demonstrate action around these risks, highlighting a focus on immediate safety [38].

It may also be that HCPs overestimate the risk to patient safety from non-treatment. Previous works show that clinicians may unconsciously inflate risks as more imminent or probable than they objectively are [39], as a result of cognitive bias. It may be that negative outcomes such as deaths are more easily recalled and therefore appear more likely due to the availability heuristic [40]. Similarly, structured risk assessments may anchor clinicians to risks [40]. Integrating these perspectives, we theorise that such cognitive tendencies can create a perceived imperative to act.

Additionally, the siloed nature of healthcare means that physical health concerns are largely handled by a separate team [41]; this separation may again shift the risk-benefit decision-making of HCPs to the risks of the psychiatric condition rather than those of medication [42]. Participants described weighing the potential harms of medication against concerns about unmanaged distress. They indicate that prescribing decisions appear to involve interpretive judgements about risk. We suggest that how individuals perceive, interpret, and quantify prescribing risks in BPD is influenced by their position within the healthcare system, expectations, and cognitive biases.

### Validation and the therapeutic relationship

Theme 3 identified the impact of the therapeutic relationship. Here, again, common sources of conflict within agency relationships are evident, in the form of divergence in goals, information and incentives. Clinicians reported seeking to establish or preserve therapeutic relationships, while patients are reported as seeking validation of their experiences. In this context, prescribing can function as a form of validation, creating incentives for clinicians to use medication to maintain engagement and trust, even when it may not align fully with their clinical judgement.

Previous studies, across a range of conditions, settings, and interventions, demonstrate that a positive therapeutic relationship correlates with positive health outcomes, including the resolution of general medical symptoms, improved functioning and other positive outcomes [43, 44].

Despite its significance, many participants reported numerous challenges in maintaining these relationships, including system constraints (described in Theme 4). Similarly, a 2020 study, exploring the therapeutic relationship in psychiatric conditions, identified multiple barriers to effective engagement, with particular emphasis on the difficulties with therapeutic relationships within “resource-challenged settings” [45]. Additionally, the difficulties in forming and maintaining well-functioning relationships faced by BPD patients, as shown in previous studies, may present particular challenges in developing therapeutic relationships [46], compounding the issue [47]. 

Given these challenges, it may be that prescribing is utilised as a facilitator of this therapeutic relationship [48]. As with previous studies, participants reflected that, in their opinion, prescribing can be validating for patients as it conveys a sense of acknowledgement and care [49], which seems to be especially true where the patient is involved in the prescribing decision [49, 50]. This would indicate that a key component of “validating treatment” is a patient’s meaningful engagement in the decision-making process. This intersection between Themes 3 and 5 underscores the importance of shared decision-making in treatment decisions; by acknowledging patient opinions, treatment can be aligned with patients’ goals. The observed levels of prescribing may therefore partly reflect HCPs’ efforts to maintain and validate the therapeutic relationship with patients [1^24^]. Across interviews, participants repeatedly emphasised the importance of sustaining the therapeutic relationship and avoiding actions that might be perceived as dismissive or invalidating. Expectation States Theory provides one view on why medication in particular is used, by suggesting that expectations based on social norms, can influence decisions [51]. Here both patients and HCPs are part of a care pathway where medication is highly visible and commonly used, as a result HCPs may assume that patients expect a prescription. These perceived expectations create a form of social pressure, HCPs anticipate that providing a prescription will meet the patients’ needs, maintain rapport, and align with professional norms. These expectation-driven interactions may contribute to higher rates of prescribing.

Furthermore the presence of a therapeutic relationship, combined with a patient’s choice to engage in a treatment, may also make the risk of prescribing more tolerable to HCPs; prescribers may feel less responsible as decision-making was shared with the patient, resulting in a belief of shared responsibility.

### System constraints

Theme 4 describes difficulties in accessing appropriate services for BPD patients. System constraints such as workforce shortages, fragmented services, and limited access to non-pharmacological interventions represent environmental constraints, consistent with Agency Theory, influencing both the options available and the degree of autonomy clinicians felt they could exercise.

Participants described, how institutional norms, such as expectations of adherence to pathways, implicitly steered decisions. These pressures often created a misalignment between HCPs, patients and organisational goals, reinforcing reliance on medication when alternative approaches are less accessible. This correlates with previous studies that have found a lack of long-term psychotherapy options for BPD patients in England [52, 53].

This may be, in part, due to the high cost of delivering long-term psychological therapies. A 2007 trial found that providing a Dialectical Behaviour Therapy (DBT) service costs £400 per-patient per-month [54], a cost far higher than that of oral medication in general. Even if cost were not a barrier to services, there are systematic shortages of trained staff to provide such therapies [51, 53].

Although not based on empirical evidence, a 2015 commentary on current clinical practices in BPD highlighted that if a range of viable alternatives were available, HCPs may be less likely to prescribe [55]. Without these alternatives, HCPs may feel reduced to a single option, given their beliefs that they need to act to reduce the risk of harm to patients from their presentation (Theme 2). Similarly, it may also be that many HCPs can prescribe medication, but few have the knowledge or capacity to provide psychological intervention. If the only tool you have available is prescribing, every problem looks like a prescribing problem.

A potential solution to this issue maybe the use of short-term psychological interventions. Although current NICE guidelines advise against their use in BPD [56], emerging evidence suggests that such interventions may play a valuable role in the care of patients with BPD [57, 58].

For instance, a 2014, 14-week group therapy programme showed sustained improvements at nine-month follow-up, despite a small sample size [59]. A 2022 Dutch qualitative study reported generally positive patient experiences with short-term mentalisation-based therapy, though some participants highlighted the need for longer-term engagement [60].

Even limited frameworks for HCPs may help in addressing this resource gap. General Psychiatric Management for Adolescents (GPM-A) has shown benefit in BPD, as a low-resource, accessible approach for clinicians without formal psychotherapeutic training [61]. While further research is needed, these new approaches present a potential to expand access and provide support for individuals with BPD.

### Patient choice

‘Patient choice’ was consistently described by participants as a key element in their decision of whether to prescribe. Though linked to the therapeutic relationship, there are unique connotations of this theme. ‘Patient choice’ represents an intrinsic source of potential conflict, given the possibility of goal divergence, differing risk perceptions, and information asymmetries regarding the role of medication in treatment. These dynamics place clinicians in the position of needing to balance patient autonomy with their professional responsibility to ensure safety and minimise harm.

In considering why patients may choose medication, participants suggest that medication is often perceived as the primary or most needed intervention. Participants frequently described medication as the treatment patients, or their carers, expected when presenting to services. It has been suggested that psychiatry increasingly risks pathologising normal behaviour [62]. Yet for patients, a diagnosis and the prescribing of medication can provide validation, shifting suffering from what might be seen as a personal failing to a recognised medical condition beyond their control, it may also facilitate access to care and other secondary benefits [63].

Moreover, it may be particularly challenging in psychiatry to refuse to provide a diagnosis, due to the Expectation States Theory and the “fuzzy boundaries” around mental health diagnosis and “normality” [62].

Furthermore, the nature of decision-making in the care of patients has evolved over time [64]; in modern healthcare, a middle ground is often advocated for, where choices are a shared decision [65]. In this model, patients have an active role in decision-making. To be an active participant, they must have information about the treatments, defining the risks and benefits in a manner tailored to local circumstances and personal preference [65, 66]. This is particularly evident in trauma-informed care pathways, a growing movement in the UK that places choice and control at the centre of treatment [32]. Such patient choice can end up in opposition to the “societal perspective” of guidelines [66], with conflict arising from differences in perspectives around the risks and benefits, evidence requirements, or other parts of decision-making [66].

In essence, while trauma-informed frameworks are reshaping practice by emphasising collaboration and patient empowerment, this also sets the stage for conflict with evidence based medicine. Such situations may be easier to navigate in cases where the risks and benefits of patients’ choices are clear. As such, in the treatment of BPD, where these are less well-defined and clinicians seem more uncertain, patient choice may hold more weight than in other fields.

### Strengths and weaknesses of the study

A major strength of this study lies in the inclusion of multiple sectors across a wide range of care providers and the inclusion of both medical and non-medical prescribers. In addition, its qualitative design and the use of interviews are often considered a staple of qualitative research [67]. This approach allowed the collection of rich and in-depth information [68] for the exploration of prescribers’ experiences in this understudied topic [13]. One key strength of this article is the relevance for day-to-day practice and the potential to improve clinical practice.

Despite this, the study had several potential limitations. The sample may not fully represent all HCPs due to potential selection bias, as it is possible that HCPs with strong experiences, opinions, or interest in the topic were more likely to engage with the project. In addition as the study did not directly observe prescribing practices, it was unable to capture any unacknowledged influences, such as unconscious biases.

Secondly, though the study benefited from substantial contributions from HCPs in primary care, the study had limited input from GPs. Although advertised to GPs, with efforts made to encourage participation, including promotion of the study by GP partners, none were able or willing to be interviewed for the study. This may reflect a belief that, as prescribing is often led by secondary care teams, GPs did not feel the need to respond to this issue.

Another key limitation of this study is its focus solely on prescribers’ views, without incorporating the voices of patients themselves. To address this, a second paper has been submitted for publication, covering the views of patients and informal carers in relation to prescribing in BPD.

Finally, while this study offers insight into prescribing for BPD, caution is required when generalising the findings. The UK context is shaped by specific regulatory frameworks and patterns of primary–secondary care integration. Different health systems, particularly those with insurance-based models, may have other significant influences beyond those in the UK.

### Future research

There is, at present, limited literature on patient decision-making in cases where treatment choices diverge from prescriber recommendations and/or are confined by resource availability. Future research should seek to develop and test an intervention using the Medical Research Council (MRC) framework for designing complex interventions [69], through, for example, the lens of relational prescribing.

Another possible area of research is the evaluation of practical tools to support the care of individuals with Borderline Personality Disorder, and their effects on prescriber patterns – be that access to brief psychological interventions for patients or training for HCPs in psychotherapeutic frameworks.

Furthermore, all interventions targeting BPD should incorporate aspects of medicines management.

### Clinical implications

This study indicates that medication is being prescribed to BPD patients for overlapping reasons. Unconscious assumptions can lead to sub-optimal clinical decisions; thus, developing an awareness of the decision-making process and the factors that influence it is essential for enhancing clinical practice. Service providers and HCPs themselves need to develop the knowledge and strategies to navigate the relational complexities of prescribing for BPD. Moreover, commissioners must offer consistent and accessible alternatives to pharmacological treatments.

## Conclusion

This study has generated a number of core themes: Symptomatic Treatment; Risk; Validation and the Therapeutic Relationship; System Constraints; and Patient Choice. These factors were identified as key influences shaping participants’ prescribing decisions. Participants highlighted the complexity of treating patients and managing risk, while maintaining a therapeutic relationship and navigating the healthcare system, indicating that prescribing decisions were often more than simple risk-benefit assessments of the prescribed pharmacological intervention. This appears to result in a high level of suboptimal prescribing.

The therapeutic relationship appears to be a central theme influencing prescribing decisions. Yet, the themes of risk, system constraints and patient choice highlight the practical dilemmas faced by HCPs in maintaining this relationship. An inability to access alternatives to pharmacological interventions and a belief in the possibility that medication can provide a positive impact, paired with concerns around potential risks, further complicate HCPs’ decision-making.

Overall, these themes provide insight into the core influences behind the high use of medication in BPD, including the relational nature of prescribing. In particular, there are indications that the presence of a therapeutic relationship, combined with a patient’s choice to engage in a treatment, may make the risks of prescribing more tolerable to HCPs.

## Supplementary Information

Below is the link to the electronic supplementary material.

Supplementary Material 1

Supplementary Material 2

## Data Availability

The interview data supporting this study will not be publicly available due to the sensitive nature of the information. Participants did not provide consent for public sharing. However, anonymised or de-identified summaries of the data, if appropriate, may be available upon request. Please contact jc517@bath.ac.uk.
