# Supplementary material for: Influences on prescribing in borderline personality disorder: insights from health care professionals: a qualitative interview study
Source: BMC Psychiatry. 2026 Apr 27;26:462. doi: 10.1186/s12888-026-08104-y (PMC13267354; doi:10.1186/s12888-026-08104-y)
Supplement: Supplementary file 1 — Supplementary Material 1 [file 12888_2026_8104_MOESM1_ESM.docx]

Appendix-A

COREQ Checklist

| **Domain 1: Research Team & Reflexivity** | | |
| --- | --- | --- |
| **Personal characteristics** | | |
| 1. Interviewer/facilitator | Which author(s) conducted the interview or focus group? | Reported on Page 6 |
| 2. Credentials | What were the researcher’s credentials? (e.g. PhD, MD) | The research team comprised JC doctoral candidate; IM (PhD); and MJ (PhD). |
| 3. Occupation | What was their occupation at the time of the study? | JC (doctoral candidate), a clinical service manager; IM Professor of Clinical Pharmacy; and MJ a Senior Lecturer. |
| 4. Gender | Was the researcher male or female? | All three researchers were male |
| 5. Experience and training | What experience or training did the researcher have? | All three researchers had received training in qualitative research methodologies, with IM and MJ possessing prior experience in applying these methods. |
| **Relationship with participants** | | |
| 6. Relationship established | Was a relationship established prior to study commencement? | Reported on Page 4 |
| 7. Participant knowledge of the interviewer | What did the participants know about the researcher? (e.g. personal goals, reasons for doing the research) | Reported on Page 4  Participants were informed that this was a research project to study influences on prescribing in BPD. |
| 8. Interviewer characteristics | What characteristics were reported about the interviewer/facilitator? (e.g. bias, assumptions, reasons and interests in the research topic) | The team had a shared research interest in the study topic and had recently co-authored a systematic review on the subject |
| **Domain 2: Study Design** | | |
| **Theoretical framework** | | |
| 9. Methodological orientation and theory | What methodological orientation was stated to underpin the study? (e.g. grounded theory, discourse analysis, ethnography, phenomenology, content analysis) | Reported on Page 6 |
| **Participant selection** | | |
| 10. Sampling | How were participants selected? (e.g. purposive, convenience, consecutive, snowball) | Reported on Page 4 |
| 11. Method of approach | How were participants approached? (e.g. face to face, telephone, mail, e-mail) | Reported on Page 4 |
| 12. Sample size | How many participants were in the study? | Table 1 |
| 13. Non-participation | How many people refused to participate or dropped out? Reasons? | NA – Participants Contacted the Research Team |
| **Setting** | | |
| 14. Setting of data collection | Where was the data collected? (e.g. home, clinic, workplace) | Reported on Page 6 |
| 15. Presence of non-participants | Was anyone else present besides the participants and researchers? | NA- No one outside of participants and researcher was present |
| 16. Description of sample | What are the important characteristics of the sample? (e.g. demographic data, date) | Table 1 |
| **Data collection** | | |
| 17. Interview guide | Were questions, prompts, guides provided by the authors? Was it pilot tested? | Reported on Page 4 |
| 18. Repeat interviews | Were repeat interviews carried out? If yes, how many? | NA – No repeat interviews were conducted |
| 19. Audio/visual recording | Did the research use audio or visual recording to collect the data? | Reported on Page 6 |
| 20. Field notes | Were field notes made during and/or after the interview or focus group? | Reported on Page 8 |
| 21. Duration | What was the duration of the interviews or focus group? | Reported on Page 6 |
| 22. Data saturation | Was data saturation discussed? | NA- Data saturation was not utilised however, the data collected was deemed to hold sufficient information power to support robust thematic analysis |
| 23. Transcripts returned | Were transcripts returned to participants for comment and/or correction? | NA- Transcript were not returned to participants |
| **Domain 3: Analysis & Findings** | | |
| **Data analysis** | | |
| 24. Number of data coders | How many data coders coded the data? | Reported on Page 6 |
| 25. Description of the coding tree | Did authors provide a description of the coding tree? | NA- No |
| 26. Derivation of themes | Were themes identified in advance or derived from the data? | Reported on Page 6 |
| 27. Software | What software, if applicable, was used to manage the data? | Reported on Page 6 |
| 28. Participant checking | Did participants provide feedback on the findings? | NA- No |
| **Reporting** | | |
| 29. Quotations presented | Were participant quotations presented to illustrate the themes/findings? Was each quotation identified? (e.g. participant number) | Reported on Page 6- 10 (Yes) |
| 30. Data and findings consistent | Was there consistency between the data presented and the findings? | Yes  Figure 1 (Yes)  Yes |
| 31. Clarity of major themes | Were major themes clearly presented in the findings? |  |
| 32. Clarity of minor themes | Is there a description of diverse cases or discussion of minor themes? |  |

Developed from: Tong A, Sainsbury P, Craig J. Consolidated criteria for reporting qualitative research (COREQ): a 32-item checklist for interviews and focus groups. *International Journal for Quality in Health Care*. 2007. Volume 19, Number 6: pp. 349 – 357
