# Supplementary material for: Influences on prescribing in borderline personality disorder: insights from health care professionals: a qualitative interview study
Source: BMC Psychiatry. 2026 Apr 27;26:462. doi: 10.1186/s12888-026-08104-y (PMC13267354; doi:10.1186/s12888-026-08104-y)
Supplement: Supplementary file 2 — Supplementary Material 2 [file 12888_2026_8104_MOESM2_ESM.docx]

Appendix B

Interview Topic Guide

This interview schedule will only be used by the Chief Investigator and will not be shared with the participants. The questions have been designed to try and ensure participants are comfortable enough to discuss their beliefs and experiences around shared prescribing decision in Borderline Personality Disorder. The schedule has been designed as a guide; the interview schedule may evolve during data collection to ensure it adequately reflects issues raised during the preceding interviews. Not all follow up prompt points will be asked to all participants.

**Introduction:**The interview will commence with personal introductions and thanking the participant for their willingness to participate in the research.

**Interview process:**The Chief Investigator will explain the intended duration of the interview, reiterate the topic, and confirm the process for signalling that an individual wishes to pause or end the interview.

**Consent:**The Chief Investigator will confirm that the appropriate version of the participants’ information leaflet has been read and understood and any remaining questions will be answered.

Consent to participate in the interview lasting up to one hour and approval to audio record and take field notes will be obtained from participants, by the Chief Investigator, beforehand and reconfirmed immediately prior to the interview.

Confidentiality, anonymity of data and the right to withdraw from the study at any time will be reiterated.

This consent will be recorded and documented.

**Dissemination of the Research Findings:**Participants will be asked if they wish to be kept informed of the research findings.

**Basic demographic information:**Participants will be asked to self-report the following information at the start of the interview:

- Ethnicity
- Age
- Sex
- Geographical location
- Years of experience
- Profession, practice setting, and role in treating patients with BPD

**INTERVIEW**

1. In general, could you tell me your experiences of treating BPD?

- Workload
- Challenges
- Support available

2. Could you tell me about your experiences of using medication for BPD?

- What medication? and why?
- How long would you use the medication?
- How do you assess if it has worked?
- Does medication change the presentation of symptoms?
- Why was this important, in terms of your prescribing decision?

3. Could you tell about your decision about prescribing in BPD?

- What makes you more or less likely to prescribe in BPD?
- Is the decision normally planned or unplanned?
- Do you discuss options?
- How involved is the patient?
- Is their a particular goal in starting medication?

4. I now want to explore what influences your prescribing in relation to BPD?

- Guidelines (or lack of them)
- Experience
- Advice from colleagues
- Patient factors (e.g., demand from patients, expectations)
- Informal carers

5. If you could identify one particular factor which influences you over the others, what would it be?

6. Is there anything else you would like to add to your answers?

**Closing**

Thank you for taking part in this study. Your interview has been audio-recorded and will be transcribed and analysed. If you have opted to receive a summary, we will contact you using the email address provided.
